# Self-Sustained Oscillatory Sliding Movement of Doublet Microtubules and Flagellar Bend Formation

**DOI:** 10.1371/journal.pone.0148880

**Published:** 2016-02-10

**Authors:** Sumio Ishijima

**Affiliations:** Department of Bioengineering, Graduate School of Bioscience and Biotechnology, Tokyo Institute of Technology, Meguro-ku, Tokyo, Japan; University of Luebeck, GERMANY

## Abstract

It is well established that the basis for flagellar and ciliary movements is ATP-dependent sliding between adjacent doublet microtubules. However, the mechanism for converting microtubule sliding into flagellar and ciliary movements has long remained unresolved. The author has developed new sperm models that use bull spermatozoa divested of their plasma membrane and midpiece mitochondrial sheath by Triton X-100 and dithiothreitol. These models enable the observation of both the oscillatory sliding movement of activated doublet microtubules and flagellar bend formation in the presence of ATP. A long fiber of doublet microtubules extruded by synchronous sliding of the sperm flagella and a short fiber of doublet microtubules extruded by metachronal sliding exhibited spontaneous oscillatory movements and constructed a one beat cycle of flagellar bending by alternately actuating. The small sliding displacement generated by metachronal sliding formed helical bends, whereas the large displacement by synchronous sliding formed planar bends. Therefore, the resultant waveform is a half-funnel shape, which is similar to ciliary movements.

## Introduction

It is now widely accepted that flagellar and ciliary movements are generated by active sliding between the adjacent doublet microtubules of the axoneme. However, information experimentally obtained on microtubule sliding is limited. Accordingly, the microtubule sliding mechanisms underlying flagellar and ciliary movements remain to be determined.

The author recently revealed that there are two types of microtubule sliding events regulated by Ca^2+^ and cAMP: (i) large sliding displacement generated by sliding synchronously throughout an extended region along one doublet and (ii) small sliding displacement generated by sliding that propagated circumferentially around the axoneme from one doublet to another along the axoneme. The first presumably corresponds to the synchronous sliding and the second the metachronal sliding [1,2]. The different ways of regulating microtubule sliding using Ca^2+^, cAMP, and MgATP^2-^ lead to various types of flagellar movements, including planar or helical bending in sea urchin spermatozoa [1]. Furthermore, these regulations generate a hyperactivated motility of mammalian spermatozoa [2].

To unambiguously prove the relationship between metachronal and synchronous sliding to flagellar and ciliary bending, it is necessary to determine directly the distribution of active sliding along each of the nine doublet microtubules at different stages of a beat cycle. To solve this problem, the author developed new sperm models that involved the use of bull spermatozoa keeping an intact motile apparatus but deprived of their plasma membrane and midpiece mitochondrial sheath [2]. When these sperm models are exposed to ATP, the fibers of the doublet microtubules bound to the outer dense fibers are extruded from the sperm flagella and form loops at the midpiece. The loop sizes change at different stages of a beat cycle. Both the sliding displacement of activated doublet microtubules and flagellar bending provide information about the active sliding of each doublet microtubule at different stages of a beat cycle and the relation of the active sliding to flagellar bending.

In the present study, the oscillatory microtubule sliding movement and the flagellar bend formation were investigated using the new sperm models at various Ca^2+^ concentrations. The doublet microtubules extruding from the sperm flagella spontaneously retracted into the sperm flagella without any flagellar bending. The synchronous and metachronal sliding coexisted in a flagellum and created a one beat cycle of flagellar bending by alternately actuating; however, these two sliding events were performed independently. The synchronous sliding produced planar bends, whereas the metachronal sliding formed helical bends. The careful observation of the disintegration of sea urchin sperm axonemes by microtubule sliding also revealed a spontaneous oscillatory sliding movement of the doublet microtubules. Based on these results, a microtubule sliding theory of flagellar movement was proposed.

## Materials and Methods

### Sperm preparations

The frozen bull semen was purchased from the National Federation of Agricultural Cooperative Associations (Tokyo, Japan). The highly motile spermatozoa were prepared using aforementioned procedures [2]. Briefly, 1.5 ml of thawed semen was layered over a gradient of 2 ml each of 45% and 90% Percoll (Sigma, Chemical Co., St Louis, MO, USA), and then centrifuged at 700 *g* for 15 min. The supernatant was removed and the sperm pellet was resuspended in fresh Tyrode’s solution devoid of CaCl_2_ (8 g NaCl, 0.2 g KCl, 0.06 g NaH_2_PO_4_·H_2_O, 0.1 g MgCl_2_·H_2_O, 1 g NaHCO_3_, and 1 g glucose per liter of deionized water; pH 7.4) to make a 0.3 ml concentrated sperm suspension.

Sea urchins *Hemicentrotus pulcherrimus* were supplied from the Tateyama Marine Laboratory of the Ochanomizu University (Tateyama, Japan). Concentrated spermatozoa of the sea urchin were obtained using an intracoelomic injection of 1.0 mM acetylcholine dissolved in artificial seawater (Jamarin U, Jamarin Laboratory, Osaka, Japan) and placed in a plastic culture dish (35 x 10 mm). The samples were stored in a refrigerator until used [1,3]. A 20 μl sample of spermatozoa was diluted with 0.2 ml Ca^2+^-free artificial seawater just before use.

### The sliding disintegration of sperm models

The sliding disintegration of bull sperm flagella was performed as aforementioned [2]. To remove the plasma membrane and the midpiece mitochondrial sheath, a 10-μl aliquot of the concentrated sperm suspension was added to 100 μl of the extraction solution (1% Triton X-100, 0.2 M sucrose, 5 mM DTT, 25 mM potassium glutamate, 0.1 mM EGTA, and 40 mM Hepes; pH 9.5) in one of the wells of a 24-well tissue culture plate and gently stirred. The mixture was then incubated for 5 min at 23°C. To examine the process of the disintegration of the sperm flagella by sliding between the adjacent doublet microtubules, an extracted sperm suspension of 10 μl was transferred to an observation chamber (0.18 mm deep, 20 mm wide, and 24 mm long) made of vinyl tape attached to the slide in two parallel strips; the trough was then covered with a glass coverslip. The flagellar disintegration by microtubule sliding was achieved by applying 150 μl of the reactivation solution (0.2 M sucrose, 1 mM DTT, 25 mM potassium glutamate, 4 mM MgSO_4_, 5 mM ATP, and 40 mM Hepes; pH 7.9) to one end of the observation chamber, whereas excess fluid was drained from the opposite end with a small pieces of filter paper. Ca^2+^-buffered reactivation solutions were used for examining the effects of Ca^2+^ on the flagellar disintegration [2,4].

The sliding disintegration of sea urchin sperm flagella was performed as aforementioned [1,3]. To remove the sperm plasma membrane with Triton X-100 and millimolar calcium (potentially symmetric condition), 10 μl of the sperm suspension was placed in one of the wells of a 24-well tissue culture plate containing 0.25 ml of extraction solution (0.15 M potassium acetate, 10 mM Tris buffer, 1 mM DTT, 0.2 mM EGTA, 0.05% (w/v) Triton X-100, 2 mM MgSO_4_, and 2 mM CaCl_2_; pH 8.2). The suspension was then gently stirred for approximately 30 s after which 10 μl of the mixture was transferred to another well containing 0.25 ml of the reactivation solution (0.25 M potassium acetate, 10 mM Tris buffer, 1 mM DTT, 2 mM EDTA or EGTA, and various concentrations of MgSO_4_, CaCl_2_, and ATP to obtain the desired Ca^2+^ or MgATP^2-^ concentrations without changing the concentrations of the other species in the solutions; pH 8.2). Approximately 20 μl of the reactivated sperm suspension was transferred to the observation chamber and the trough was covered with a glass coverslip. Microtubule sliding was achieved by applying a small volume of the reactivation solution containing 20 μg/ml elastase (E-0127; Sigma Chemical Co., St. Louis, MO, USA) to one end of the observation chamber and excess fluid was drained from the opposite end with small pieces of filter paper.

### Data recording and analysis

The disintegration of the sperm flagella was recorded using a Nikon Eclipse E600 microscope equipped with a phase-contrast condenser and 100x DL objective for bull spermatozoa and with a dark-field condenser and 40x objective for sea urchin spermatozoa. Images were captured directly into the computer hard-drive using a Panasonic CCD video camera (WV-BL 730, Matsushita Communication Industrial Co., Ltd., Yokohama, Japan), image software (Dipp-Motion 2D, Ditect Co., Ltd., Tokyo, Japan) and a frame grabber (SIM-PCI, Ditect Co., Ltd.) at the rate of 60 images per second.

Sliding displacement was defined as the difference in length between adjacent fibers, measured using the Autotrace module of Bohboh software (Media Land Co., Ltd., Tokyo, Japan) [5].

### Statistical analysis

All data are expressed as the mean ± s.d. Data were analyzed using one-way ANOVA with Scheffe’s post-hoc test using SPSS 11.0J (SPSS Japan Inc., Tokyo). The level was considered significant with *P*<0.05.

## Results and Discussion

### The spontaneous oscillatory sliding movement of doublet microtubules in the bull sperm models

When the plasma membrane and the midpiece mitochondrial sheath of bull spermatozoa are removed and exposed to ATP, more than 40% of the sperm models normally beat for a period, stop bending, and then begin to disintegrate due to sliding of the doublet microtubules [2]. Most fibers extruded from the sperm flagella (more than 90%, Table 1), composed of the doublet microtubules and the outer dense fibers, stop sliding and form two types of loops at the midpiece: a long and thick loop and short and thin loop. The long and thick loop is extruded by synchronous sliding along the flagellum, whereas the short and thin loop is generated by propagation sliding from one doublet to another [1,2].

**Table 1:** Ca^2+^ Dependency on the Occurrence Rate of the Oscillatory Microtubule Sliding Movement.

Ca^2+^ concentration (M)	10^−5^	10^−7^	10^−8^	10^−9^
**Occurrence rate (%)**	2.2 ± 1.1 (1946, 6)	2.8 ± 1.4 (1087, 5)	5.4 ± 1.8 (638, 5)	8.1 ± 3.5 (339, 6)

Data are presented as mean ± s.d. Numbers in parentheses represent the total sperm scored and the number of experiments performed. Significant differences were found between values at different Ca^2+^ concentrations except between 10^−5^ and 10^−7^ M.

**Fig 1:**
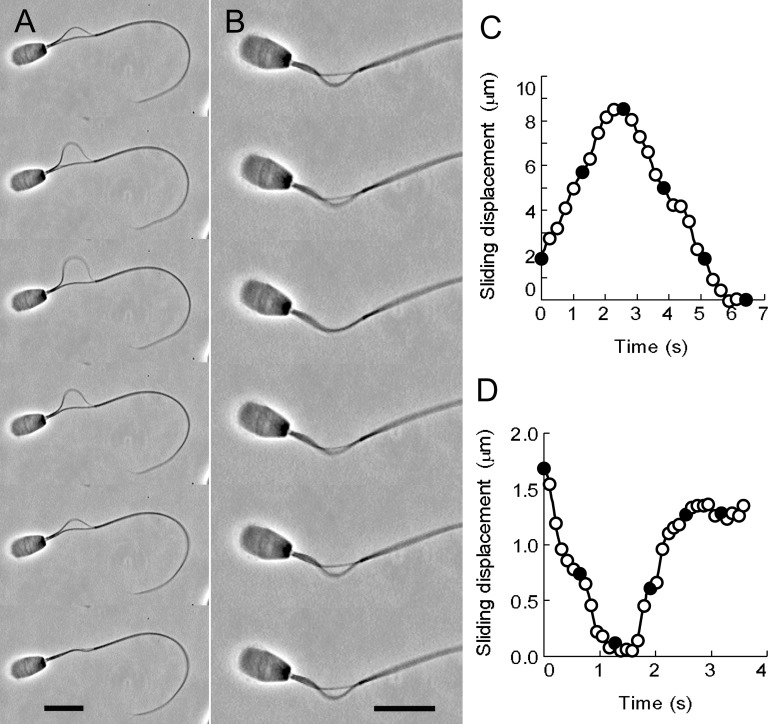
The oscillatory sliding movement of the doublet microtubules of bull sperm models. (A) Phase-contrast video micrographs showing the oscillatory sliding movement of the doublet microtubules extruded by synchronous sliding. (B) Phase-contrast video micrographs showing the oscillatory sliding movement by metachronal sliding. The free-Ca^2+^ solution concentration was adjusted to 10^−9^ M. Time interval between successive images is 1.28 s in (A) and 0.64 s in (B). The profiles of the microtubule sliding displacement of A and B are shown in (C) and (D), respectively. The sliding velocity of the synchronous sliding movement was 3.02 μm/s, which was calculated between frames 1 and 2 in A (C). The sliding velocity of the metachronal sliding movement was 1.23 μm/s between frames 1 and 3 in B (D). Filled circles in C and D are the values of the sliding displacement obtained from the frames shown in A and B. Bars = 10 μm.

**Fig 2:**
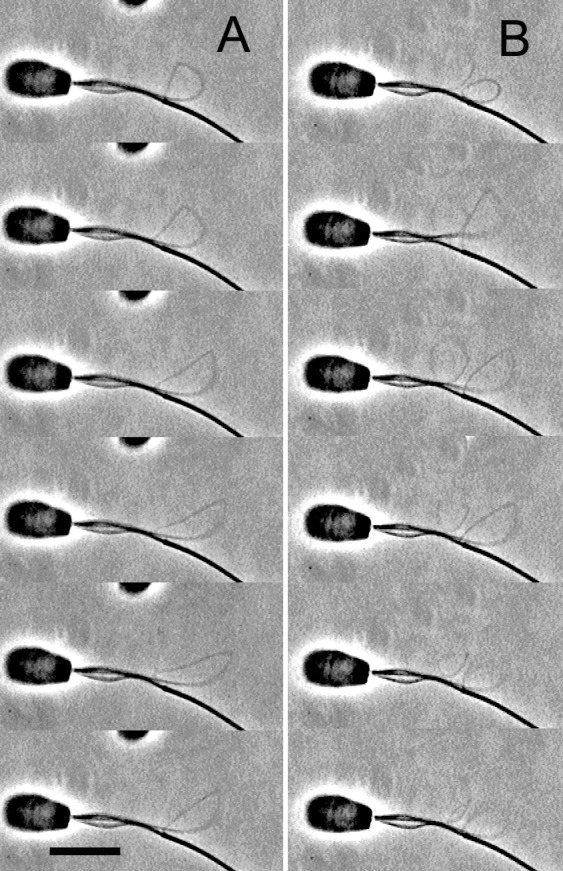
The oscillatory sliding movement of the doublet microtubules having a different configuration of bull sperm models. Phase-contrast video micrographs showing the oscillatory sliding movements of the doublet microtubules extruded by synchronous sliding. The free-Ca^2+^ solution concentration was adjusted to 10^−9^ M. Time interval between successive images is 0.75 s in (A) and 0.72 s in (B). (A and B) The same spermatozoon. The oscillatory sliding movement of a fiber having a strophoid curve (A). After the oscillatory sliding movement of the fiber for approximately 5 minutes, a new fiber was extruded from the axoneme and then began its oscillatory sliding movement (B). Bar = 10 μm.

In the current study, some of the fibers (less than 10%, Table 1) extruding from the sperm flagella spontaneously retracted into the flagella (Figs 1, 2A and 2B, S1–S3 Movies). Both the long and thick loop generated by synchronous sliding and the short and thin loop generated by metachronal sliding gradually disappeared; however, their sliding profiles were quite different; namely, the sliding displacement of the synchronous sliding movement was usually much larger than that of the metachronal sliding movement, and the sliding velocity of the synchronous sliding movement was higher than that of the metachronal sliding movement (Fig 1C and 1D). This spontaneous oscillatory sliding movement of microtubule fibers usually continued for more than 100 cycles. The occurrence rate of the oscillatory sliding movement was negatively correlated with Ca^2+^ concentrations (Table 1), suggesting that the sliding between many pairs of adjacent doublet microtubules at various loci on a flagellum is necessary for the retraction of the doublet microtubules because the number of fibers extruded from the axoneme increases at low Ca^2+^ concentrations (Fig 2, S2 and S3 Movies) [1,2]. Flagellar bending was not necessary for the retraction of the microtubule fibers (Figs 1, 2A and 2B), demonstrating that the oscillatory sliding movement is regulated by the microtubule sliding itself and not by other bending parameters such as the curvature of flagellar bends.

**Fig 3:**
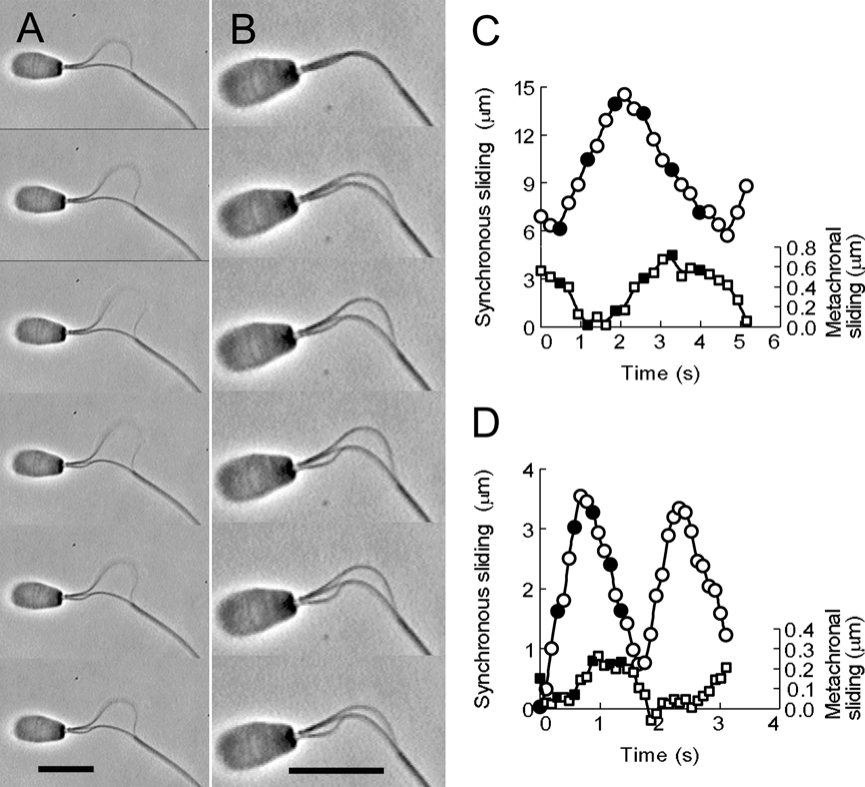
Coordination between the synchronous and metachronal sliding movements of the doublet microtubules of bull sperm models. (A and B) Phase-contrast video micrographs showing two distinct oscillatory sliding movements of the doublet microtubules by synchronous and metachronal sliding on a flagellum. Free-Ca^2+^ solution concentration was adjusted to 10^−9^ M. Time interval between successive images is 0.70 s in (A) and 0.29 s in (B). The profiles of the microtubule sliding displacement of A are shown in (C). The profiles of two cycles of the microtubule sliding displacement of B are shown in (D). Sliding velocity was 5.58 μm/s between frames 1 and 3 in A for the synchronous sliding movement, and 0.40 μm/s between frames 3 and 5 in A for the metachronal sliding movement (C). Circles represent values of the sliding movement of the doublet microtubules extruded by the synchronous sliding, whereas the values shown as squares represent the metachronal sliding. Filled circles and squares in C and D are values of the sliding displacement obtained from the frames shown in A and B. Bars = 10 μm.

The coexistence of synchronous sliding with metachronal sliding on a flagellum was sometimes observed (35.3 ± 13.4% of the oscillatory microtubule sliding, mean ± s.d. of 391 sperms in six different experiments at 10^−9^ M Ca^2+^, Fig 3A and 3B). In these cases, metachronal sliding usually appeared on the opposite side of the midpiece. Accordingly, the sperm flagella had separated into three distinct strands (Fig 3, S4 Movie), probably due to the attachment of doublet microtubules Nos. 3 and 8 to the fibrous sheath partitions of the flagellum into two compartments: a smaller one containing doublet microtubules Nos. 9, 1, and 2 and a larger one containing doublet microtubules Nos. 4, 5, 6, and 7 [6,7]. These specimens served to clarify the relationship between the synchronous and metachronal sliding movement. When the fiber of the doublet microtubules from the anterior end of the fibrous sheath was extruded by synchronous sliding, the one from the base of the midpiece that was extruded by metachronal sliding retracted into the flagella (frames 1–3 in Fig 3A and 3C). Moreover, when the fiber of the doublet microtubules synchronously sliding retracted into the fibrous sheath, the fiber at the base of the midpiece was extruded by metachronal sliding (frames 4–6 in Fig 3A and 3C). In other words, the synchronous and metachronal sliding was alternately actuated (S4 Movie); however, the total amount of sliding displacement and sliding velocity were fundamentally different between these two sliding mechanisms (Fig 3C), demonstrating clearly that synchronous and metachronal sliding are generated by the different pairs of doublet microtubules, as aforementioned. Furthermore, another set of well-focused and enlarged images of the synchronous and metachronal sliding movements showed similar characteristics pertaining to oscillatory sliding (Fig 3B and 3D).

### Coordination between synchronous and metachronal sliding and flagellar bend formation

**Fig 4:**
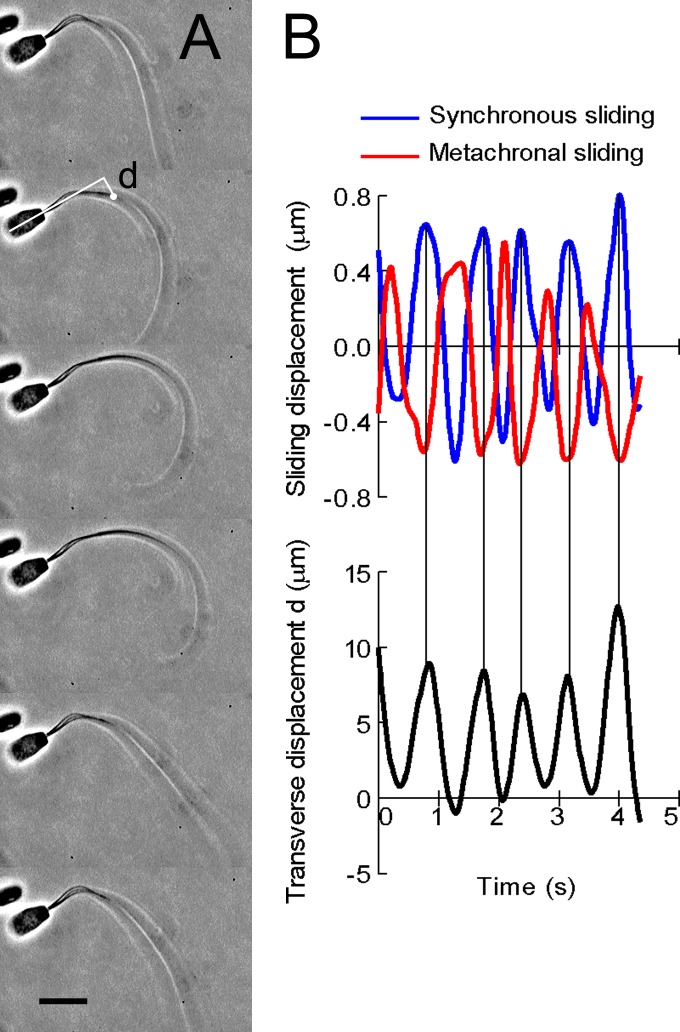
The association of microtubule sliding with flagellar bending of bull sperm models. (A) Phase-contrast video micrographs showing oscillatory synchronous and metachronal sliding and flagellar bending. The first cycle of flagellar bending is shown. The free-Ca^2+^ solution concentration was adjusted to 10^−9^ M. Time interval between successive images is 0.15 s. The beat frequency of flagellar bending was 1.2 Hz. (B) The profiles of five cycles of the sliding displacement of synchronous and metachronal sliding movements and of the transverse displacement of the flagellum shown in A. The transverse displacement of sperm flagellum from the sperm head axis (d) was measured at a position 15 μm from the head-tail junction (A). Bar = 10 μm.

**Fig 5:**
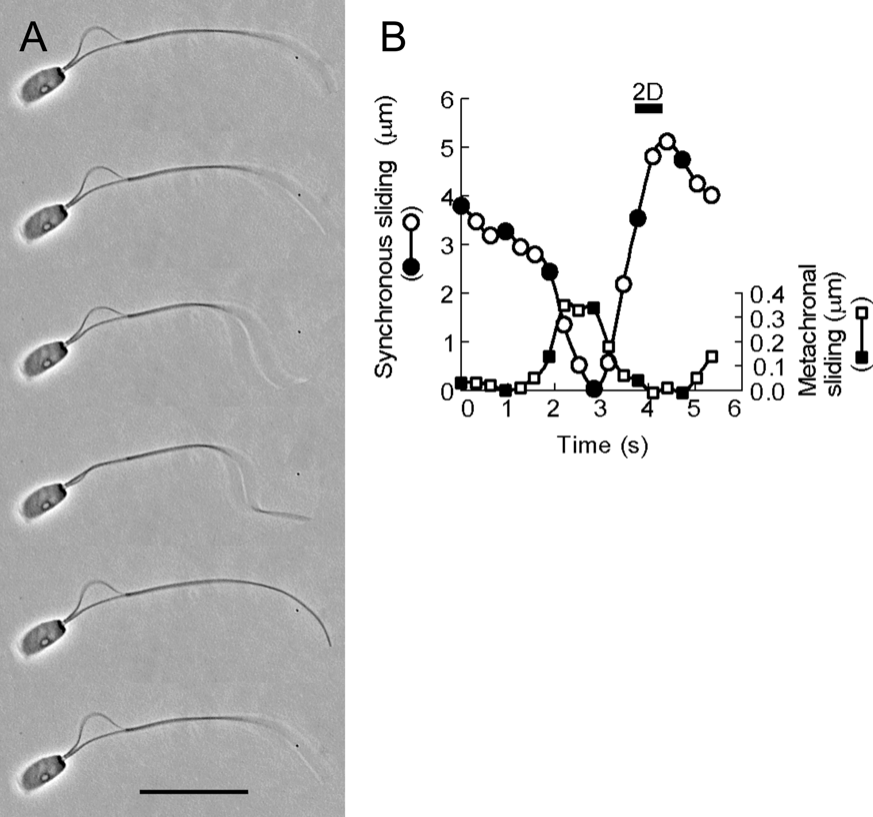
The association of microtubule sliding with flagellar waveform of bull sperm models. (A) Phase-contrast video micrographs showing oscillatory synchronous and metachronal sliding and flagellar bending. A left-handed helical bend at a distal region of the flagellum was determined by differential focusing: the flagellar parts were focused on the higher focal plane. The free-Ca^2+^ solution concentration was adjusted to 10^−9^ M. Time interval between successive images is 0.94 s. The beat frequency of flagellar bending was 0.18 Hz. (B) The profiles of the sliding displacement of synchronous and metachronal sliding movements shown in A. Circles represent the synchronous sliding values and squares the metachronal sliding values. The bar denoted by letters “2D” expresses the duration of the planar flagellar bending. Filled circles and squares in B are values of the sliding displacement obtained from the frames shown in A. Bar = 20 μm.

**Fig 6:**
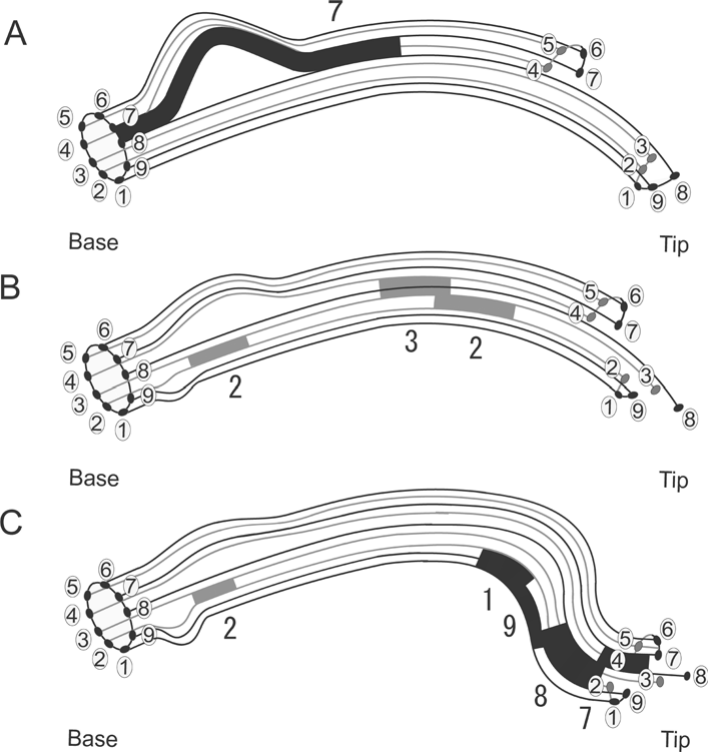
Hypothetical diagrams explaining flagellar bend formation from two distinct oscillatory microtubule sliding events. (A) Synchronous sliding between the doublet Nos. 7 and 8 generates a large fiber loop of the doublet microtubule Nos. 4–7 at the midpiece and forms an asymmetrical planar bend. The sliding displacement of the synchronous sliding is dependent on the Ca^2+^ concentration. Higher Ca^2+^ concentrations produce larger sliding displacements [1,2,4]. (B) Metachronal sliding between the doublet Nos. 3 and 4 retracts the extruded fiber of the doublets Nos. 4–7 into the flagellum, followed by metachronal sliding between the doublets Nos. 2 and 3 in two loci on the flagellum. The sliding at the base of the midpiece generates a small fiber loop for the doublets Nos. 9–2 and the sliding at the distal region of the flagellum begins to induce metachronal sliding between the doublets Nos. 1 and 2. (C) Metachronal sliding propagating towards the tip and simultaneously transferring in the sequence of doublets Nos. 1, 9, 8, and 7 leads to the generation of a left-handed helical bend at a distal region of the flagellum at low Ca^2+^ concentrations [1,8,9]. The numbers in the circles indicate the number of the doublet microtubules according to Afzelius [11]. The numbers indicate the sliding of corresponding doublet microtubules. The synchronous sliding superimposed on the metachronal sliding converts a helical bend into a half-funnel shape.

To investigate the relation of the synchronous and metachronal sliding to flagellar bending, the flagellar bending of spermatozoa that maintained oscillatory sliding at the midpiece was analyzed. One cycle of the oscillatory sliding movement was closely related with that of the flagellar bending movement (Fig 4, S5 Movie). Another set of well-focused images of the sliding movement and flagellar bending showed that a propagating three-dimensional bend was observed at the distal regions of a flagellum during most parts of the beat cycle, and a big shift from the focal plane was generated by the metachronal sliding (frames 3 and 4 in Fig 5A and 5B). This finding is consistent with previous results showing that metachronal sliding is a short sliding transfer event from one doublet to another [1,2]. In contrast, a planar bend well focused over the entire length of a flagellum was observed for a short time frame during the beat cycle (approximately 1/10 of a beat cycle, frame 5 in Fig 5), which corresponded to the maximum extrusion of the fiber by synchronous sliding (Fig 5B). These observations demonstrate clearly that planar bends are generated by synchronous sliding. Based on these results, the hypothetical microtubule sliding mechanisms of flagellar movement were constructed (Fig 6). Fundamentally, metachronal sliding propagates from the base to the tip along the sperm flagellum and at the same time transfers from doublet to doublet around the axoneme, generating helical bends [1,8,9]. During a cycle of this metachronal sliding movement, the synchronous sliding between a specific pair of doublet microtubules, probably doublets Nos. 7 and 8 [6,10,11], occurs in a Ca^2+^ concentration-dependent way and generates planar bends (Fig 6A) [1,2,4]. Thus, synchronous sliding superimposed on metachronal sliding modifies the helical bends into half-funnel shapes. These shapes of flagellar bending were also observed in human spermatozoa [12].

### The spontaneous reversal of the sliding direction of the doublet microtubules extruded from sea urchin sperm axonemes

**Fig 7:**
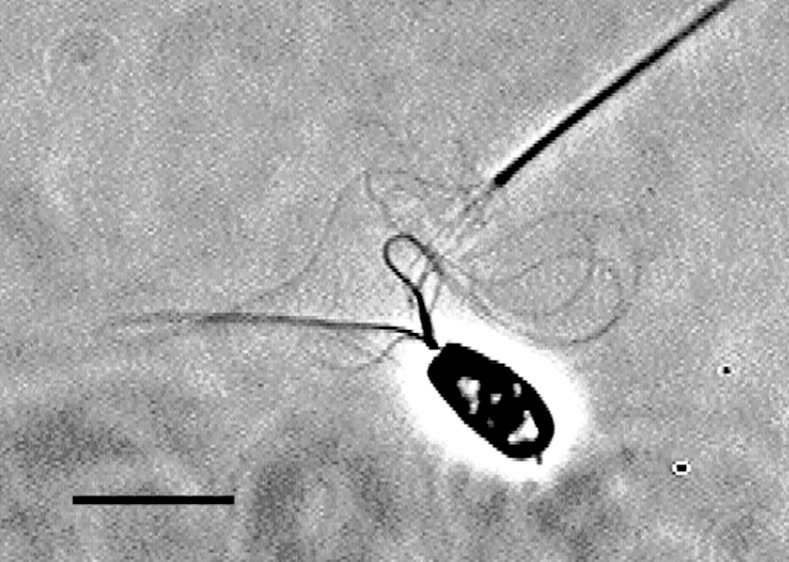
Sliding disintegration of elastase-treated axonemes of bull sperm models. A phase-contrast video micrograph showing sliding disintegration of an elastase-treated bull sperm axoneme. Elastase of 20 μg/ml was added to the reactivation solution. The free-Ca^2+^ solution concentration was adjusted to 10^−9^ M. Bar = 10 μm.

When the bull sperm models that had been deprived of plasma membrane and mitochondria were exposed to ATP and elastase, the sperm flagella essentially disintegrated into doublet microtubules, and the extruded fibers did not retract into the flagella (Fig 7). However, under the extraction conditions used in the present study, the nexin links are not extracted and remain intact at the bull sperm axoneme [6,13]. This suggests that the nexin links, preventing the disintegration of sperm axonemes, are essential in generating the oscillatory sliding movement in bull sperm models. However, the total sliding distance of the doublet microtubules observed in the present study is significantly larger than the nexin links extension, particularly during the synchronous sliding.

**Fig 8:**
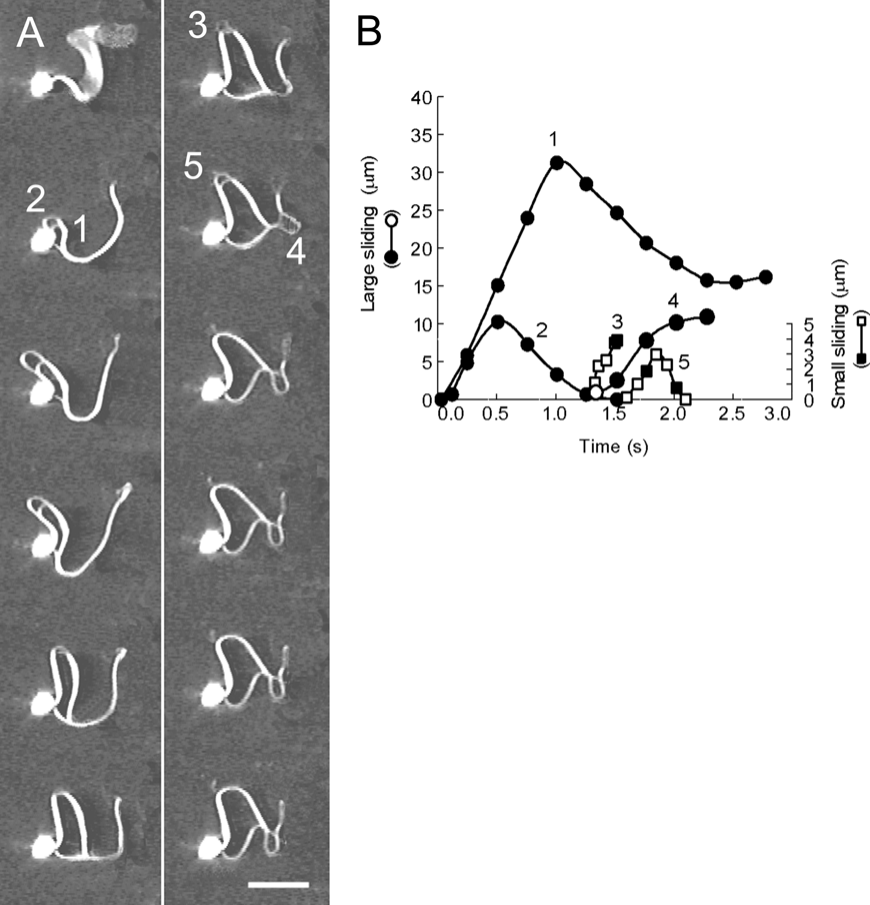
The spontaneous reversal of the sliding direction of the doublet microtubules of sea urchin sperm axonemes. (A) Dark-field video micrographs showing the sliding movements of the doublet microtubules. The sea urchin sperm axoneme still beats in the first frame. The microtubule sliding was induced with 0.5 mM MgATP^2-^ and 20 μg/ml elastase. The numbers indicate the microtubule sliding events that occur on a flagellum. The free-Ca^2+^ solution concentration was adjusted to 10^−9^ M. Time interval between successive images is 0.25 s. (B) The profiles of microtubule sliding displacement shown in A. The numbers correspond to those of the microtubule sliding shown in A. The circles represent values of large sliding displacements and squares represent small sliding displacements. The sliding velocity was 31.9 μm/s, as calculated between frames 1 and 5 of the sliding No. 1 and 24.5 μm/s between frames 1 and 3 of the sliding No. 2. Filled circles and squares are values of the sliding displacement obtained from the frames shown in A. Bar = 15 μm.

The direct observation of the oscillatory sliding movement of doublet microtubules in the bull sperm models justifies examining the oscillatory sliding of the doublet microtubules of sea urchin sperm axonemes because the sea urchin sperm axonemes take several seconds for the complete disintegration by ATP and elastase. When the sea urchin spermatozoa extracted from the plasma membrane were exposed to ATP and elastase in a low Ca^2+^ concentration solution, the fibers of the doublet microtubules were extruded from the sperm axonemes and then spontaneously retracted into the axonemes over a short period of time before the complete disintegration of the sperm axonemes (Fig 8). During this retraction into the axonemes, there was a negligible change in the curvature of the flagellar bend, demonstrating that the retraction of the doublet microtubules occurs by microtubule sliding in the sea urchin spermatozoa as well. The microtubule sliding observed under these conditions was mainly metachronal sliding, namely, the short and thin fibers of the doublet microtubules occurred between many pairs of adjacent doublet microtubules at different loci of the flagellum (Fig 8) [1]. This finding correlates well with the microtubule sliding found to generate the helical bends of demembranated and reactivated sperm flagella at low Ca^2+^ concentrations [1]. Importantly, these results suggest that nexin links play a central role in the regulation of oscillatory flagellar movement of spermatozoa, i.e., they restrict the sliding displacement of the doublet microtubules, particularly during metachronal sliding and convert the microtubule sliding into flagellar bending. The sliding velocity of the doublet microtubules of sea urchin spermatozoa was noticeably higher than that of bull spermatozoa (Fig 8). The difference in velocity may result from the large difference in the stiffness of the extruded fibers owing to the tight binding of the outer dense fibers to the doublet microtubules in bull sperm models [14].

## Supporting Information

S1 Movie The oscillatory sliding movement of the fibers extruded from the bull sperm axoneme. (AVI)

S2 Movie The oscillatory sliding movement of a fiber having a strophoid curve of bull sperm axoneme. (AVI)

S3 Movie The oscillatory sliding movement of two fibers having a strophoid curve of bull sperm axoneme. (AVI)

S4 Movie Coordination between the synchronous and metachronal sliding movements of the fibers extruded from bull sperm axoneme. (AVI)

S5 Movie The association of microtubule sliding with flagellar bending of bull sperm axoneme. (AVI)

## References

[1] Ishijima S. Regulations of microtubule sliding by Ca^2+^ and cAMP and their roles in forming flagellar waveforms. Cell Struct Funct. 2013;38: 89–95. 23546177 10.1247/csf.12021

[2] Ishijima S. Ca^2+^ and cAMP regulations of microtubules sliding in hyperactivated flagellar movement of bull spermatozoa. Proc Jpn Acad Ser B 2015;91: 99–108. 25765012 10.2183/pjab.91.99 PMC4410089

[3] Ishijima S, Kubo-Irie M, Mohri H, Hamaguchi Y. Calcium-dependent bidirectional power stroke of the dynein arms in sea urchin sperm axonemes. J Cell Sci. 1996;109: 2833–2842. 9013331 10.1242/jcs.109.12.2833

[4] Ishijima S, Mohri H, Overstreet JW, Yudin AI. Hyperactivation of monkey spermatozoa is triggered by Ca^2+^ and completed by cAMP. Mol Reprod Dev. 2006;73: 1129–1139. 16804884 10.1002/mrd.20420

[5] Ohmuro J, Ishijima S. Hyperactivation is the mode conversion from constant-curvature beating to constant-frequency beating under a constant rate of microtubule sliding. Mol Reprod Dev. 2006;73: 1412–1421. 16894536 10.1002/mrd.20521

[6] Olson GE, Linck RW. Observations of the structural components of flagellar axonemes and central pair microtubules from rat sperm. J Ultrastruct Res. 1977;61: 21–43. 915974 10.1016/s0022-5320(77)90004-1

[7] Kanouse KS, Casey C, Lindemann CB. Inhibition of microtubule sliding by Ni^2+^ and Cd^2+^: Evidence for a differential response of certain microtubule pairs within the bovine sperm axoneme. Cell Motil Cytoskeleton 1993;26:66–76. 8221908 10.1002/cm.970260107

[8] Ishijima S, Hamaguchi MS, Naruse M, Ishijima SA, Hamaguchi Y. Rotational movement of a spermatozoon around its long axis. J Exp Biol. 1992;163: 15–31. 1556511 10.1242/jeb.163.1.15

[9] Ishijima S, Hamaguchi Y. Calcium ion regulation of chirality of beating flagellum of reactivated sea urchin spermatozoa. Biophys J. 1993;65: 1445–1448. 8274638 10.1016/S0006-3495(93)81210-4 PMC1225871

[10] Sale WS. The axonemal axis and Ca^2+^-induced asymmetry of active microtubule sliding in sea urchin sperm tails. J Cell Biol. 1986;102: 2042–2052. 2940250 10.1083/jcb.102.6.2042 PMC2114254

[11] Afzelius BA. Electron microscopy of the sperm tail. Results obtained with a new fixative. J Biophys Biochem Cytol. 1959;5: 269–278. 13654448 10.1083/jcb.5.2.269 PMC2224653

[12] Ishijima S, Oshio S, Mohri H. Flagellar movement of human spermatozoa. Gamete Res. 1986;13: 185–197.

[13] Smithwick EB, Young LG. Ultrastructural evaluation of the isolation of mitochondria-free bull sperm flagella. Biol Reprod. 1978;19: 280–290. 214177 10.1095/biolreprod19.2.280

[14] Ishijima S. The velocity of microtubule sliding: its stability and load dependency. Cell Motil Cytoskeleton 2007;64: 809–813. 17685439 10.1002/cm.20228

